# *Portulaca oleracea L*. Extract Ameliorates Intestinal Inflammation by Regulating Endoplasmic Reticulum Stress and Autophagy

**DOI:** 10.1002/mnfr.202100791

**Published:** 2022-01-09

**Authors:** Ziwei Zhang, Dan Qiao, Yali Zhang, Qian Chen, Yujun Chen, Yingjue Tang, Renye Que, Ying Chen, Lie Zheng, Yancheng Dai, Zhipeng Tang

**Affiliations:** ^1^ Institute of Digestive Diseases LongHua Hospital Shanghai University of Traditional Chinese Medicine Shanghai 200032 China; ^2^ Department of Gastroenterology Shanghai Traditional Chinese Medicine‐Integrated Hospital Shanghai University of Traditional Chinese Medicine Shanghai 200082 China; ^3^ Department of Gastroenterology Traditional Chinese Medicine Hospital of Shaanxi Province Xi'an 730000 China

**Keywords:** autophagy, endoplasmic reticulum stress, IL‐10^–/–^ mice, inflammatory bowel disease, intestinal epithelial cells, *Portulaca oleracea L*

## Abstract

**Scope:**

To investigate the role of endoplasmic reticulum stress (ERS)‐induced autophagy in inflammatory bowel disease (IBD) and the intervention mechanism of *Portulaca oleracea L*. (POL) extract, a medicinal herb with anti‐inflammatory, antioxidant, immune‐regulating, and antitumor properties, in vitro and in vivo.

**Methods and Results:**

An IL‐10‐deficient mouse model is used for in vivo experiments; a thapsigargin (Tg)‐stimulated ERS model of human colonic mucosal epithelial cells (HIECs) is used for in vitro experiments. The levels of ERS‐autophagy‐related proteins are examined by immunofluorescence and Western blot. Cellular ultrastructure is assessed with transmission electron microscopy.

POL extract promotes a healing effect on colitis by regulating ERS‐autophagy through the protein kinase R‐like endoplasmic reticulum kinase (PERK)‐eukaryotic initiation factor 2α (eIF2α)/Beclin1‐microtubule‐associated protein light chain 3II (LC3II) pathway.

**Conclusion:**

Overall, the results of this study further confirm the anti‐inflammatory mechanism and protective effect of POL extract and provide a new research avenue for the clinical treatment of IBD.

## Graphical abstract

ERS‐induced autophagy is involved in inflammation, intestinal mucosal barrier damage, and pathogenesis of IBD. POL extract exerts a therapeutic effect in PAC IL‐10^‐/‐^ model and Tg‐stimulated ERS model of HIECs by regulating the PERK‐eIF2α/Beclin1‐LC3II pathway.

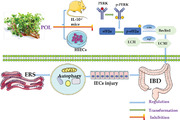

## 1 Introduction

Inflammatory bowel disease (IBD) is a chronic, non‐specific inflammatory condition of the gastrointestinal (GI) tract, including ulcerative colitis (UC) and Crohn's disease (CD).^[^ ^1^ ^]^ The disease is quite common in Western countries, with a prevalence rate of 100–2200/100 000 in Europe and North America. The number of reported cases in China has significantly increased in recent years.^[^ ^2^, ^3^ ^]^ According to the current epidemiological survey and available statistical data, the number of IBD cases in China is estimated to increase to 1.5 million by 2025. Disease incidence is relatively common in young people and has a considerable impact on social productivity and personal quality of life.^[^ ^4^ ^]^

Recent studies have found that the disruption of intestinal mucosal barrier homeostasis induced by the interaction of genetic, infectious, immune, and environmental factors is the core event causing the onset and progression of IBD. Damage to intestinal epithelial cells (IECs), an important component of the intestinal mucosal barrier, may play a crucial mediating role in this event.^[^ ^5^, ^6^ ^]^ IECs have a well‐developed endoplasmic reticulum structure, and they are one of the most active cell groups. Sustained and severe endoplasmic reticulum stress (ERS)‐induced autophagy in IECs may be the main cause of inflammation, intestinal mucosal barrier damage, and pathogenesis of IBD.^[^ ^7^, ^8^, ^9^ ^]^

*Portulaca oleracea L*. (purslane, POL) is a medicinal herb with anti‐inflammatory, antioxidant, immune‐regulating, and antitumor activities.^[^ ^10^ ^]^ Traditional Chinese medicine suggests that POL can clear heat and detoxify the body, cool blood, stop bleeding, and stop dysentery. POL is commonly used to treat bacterial dysentery, colitis, acute gastroenteritis, acute appendicitis, and hemorrhoids.^[^ ^11^ ^]^ In addition, POL has a potential repair effect on intestinal inflammation and intestinal epithelial cell injury. POL can be used to treat dextran sulfate sodium (DSS)‐induced colitis by inhibiting the oxidative stress response of malondialdehyde, nitric oxide, and superoxide dismutase, decreasing the expression of pro‐inflammatory cytokines tumor necrosis factor–α (TNF‐α), interleukin (IL)‐1β, and IL‐6 mRNA and the expression of TNF‐α and nuclear factor kappa‐B (NF‐κB) p65 protein and restoring peroxisome proliferator‐activated receptor γ levels.^[^ ^12^, ^13^, ^14^ ^]^ Most scholars have studied the mechanism of POL in the treatment of colitis through DSS‐induced colitis in mouse models.^[^ ^15^ ^]^ However, the DSS‐induced colitis model has a short maintenance time and self‐healing properties. For the chronic IBD model, repeated DSS stimulation is required to maintain inflammatory symptoms.^[^ ^16^ ^]^ Compared with the DSS‐induced colitis model, the piroxicam‐accelerated colitis IL‐10‐deficient (PAC IL‐10^–/–^) mouse model integrates dysfunction of both the intestinal barrier and the immune system. After the withdrawal of the drug, PAC IL‐10^–/–^ mice experience a chronic disease course without self‐healing. Therefore, this model is better and of great significance for further exploration of IBD.^[^ ^17^, ^18^ ^]^

The aim of this study was to investigate the role of ERS‐induced autophagy in IBD and the intervention mechanism of POL extract in vivo (PAC IL‐10^–/–^ model) and in vitro (thapsigargin (Tg)‐stimulated ERS model of human colonic mucosal epithelial cells (HIECs)).

## 2 Experimental Section

### 2.1 Preparation of POL Lyophilized Powder and 5‐Amino Salicylic Acid

The dried aerial parts of POL were triturated into powder and boiled at 80 °C for 60 min in distilled water (25 g powder per 120 mL). Then, the mixture was shaken for three cycles (15 s per cycle) with an ultrasonic shaker and filtered with a 2 mm filter. The filtrates were then dried using a freeze dryer, and the resulting lyophilized POL powder was stored at ‐20 °C. 5‐Amino salicylic acid (5‐ASA; H19980148, Jiamusi Luling Pharmaceutical Co., Ltd., China), the most commonly prescribed drug for IBD, was used as a positive control drug. The dosage administered to mice was 100 mg kg^−1^ d^−1^, chosen based on a previous study.^[^ ^19^ ^]^

### 2.2 Ultra‐Performance Liquid Chromatography Quadrupole‐Time of Flight Mass Spectrometer (UPLC‐Q‐TOF/MS)

Fifty milligrams of POL powder were placed in centrifugation tubes, and 1 mL of methanol was added. This solution was sonicated twice to aid dissolution (300 W, 40 kHz) and centrifuged at 12 000 rpm for 5 mins. UPLC analysis was conducted by UPLC/Q‐TOF‐MS (Sciex Triple TOF 4600 high resolution mass spectrum coupled with an Agilent 1290 UPLC system; AB Sciex, Darmstadt, Germany; Agilent, Los Angeles, CA, USA). Three microliters of supernatant were injected into the UPLC system equipped with an Acquity UPLC HSS T3 column (2.1 × 100 mm, 1.8 µm; Waters, Milford, CT, USA) for separation. Gradient elution was performed at a flow rate of 0.3 mL min^−1^ using the following solvent system: 0.1% formic acid‐water A), 0.1% formic acid‐acetonitrile B); 5% B 0–5 min; 5% B‐95% B from 5 to 35 min (**Table S1**, Supporting Information).

### 2.3 Animal Model

A total of 6 C57BL/6 IL‐10^–/–^ mice were purchased from Shanghai Research Center for Southern Model Organisms (No. SCXK (Shanghai) 2017–0010). The mice were raised and bred at the Animal Experiment Center of Shanghai University of Traditional Chinese Medicine (SPF grade) with controlled temperature (23±3°C), humidity (35%‐45%), and lighting (12 h d^−1^). The mice were provided with food and water ad libitum. The genes of offspring were tested by Shanghai Research Center for Southern Model Organisms. All animal studies (including the mouse euthanasia procedure) were performed in compliance with the regulations and guidelines of Shanghai University of Traditional Chinese Medicine institutional animal care and conducted according to the AAALAC and IACUC guidelines (PZSHUTCM191108003).

Male and female IL‐10^–/–^ mice of the F6 generation (1:1) tested by Shanghai Research Center for Southern Model Organisms were randomly divided into four groups (six mice for each group): the control group, piroxicam group, 5‐ASA group (100 mg kg^−1^ d^−1^) and POL group (400 mg kg^−1^ d^−1^). The body weights of the mice were recorded on a daily basis. A disease activity index (DAI) score was calculated according to a described method to assess disease severity (**Table** **1**).^[^ ^20^ ^]^ At the end of the experiments, the mice were sacrificed, and the colon tissues from the ileocecal junction to the anal verge were collected. The colon tissues were visually observed and measured. Two 0.5 cm distal colon segments for all mice were used for HE staining and TEM observation respectively. The remaining colon were stored at ‐80 °C and used for western blotting.

**Table 1:** Scoring system for DAI

Score	Weight loss [%]	Stool consistency	Gross bleeding
0	0	Normal	Negative
1	1–5		
2	6–10	Loose	Hemoccult positive
3	11–15		
4	>15	Diarrhea	Bleeding

* DAI = (score of weight loss + stool consistency + gross bleeding)/3.

### 2.4 Dosage Information

Male and female IL‐10^–/–^ mice were randomly divided into four groups: the control group, piroxicam group, 5‐ASA group (100 mg kg^−1^ d^−1^) and POL group (400 mg kg^−1^ d^−1^). The piroxicam group, 5‐ASA group, and POL group were administered 200 ppm piroxicam (P0847, Sigma‐Aldrich) in feed for 14 d to induce colitis, while the control group was given normal mouse chow.^[^ ^21^ ^]^ Simultaneously, the 5‐ASA and POL groups were given 5‐ASA and POL, respectively, by gavage starting on the first day. The control and piroxicam groups were administered equal doses of normal saline. Another six wild‐type C57BL/6 mice served as the wild‐type group. According to the Meeh‐Rubner formula, the conversion coefficient between human and mouse doses is 1:8,^[^ ^22^ ^]^ the mice dose of 5‐ASA and POL equates to human dose of 12.5 and 50 mg kg^−1^ d^−1^. For an individual with a body mass of 60 kg, to achieve this dose they would need to consume 0.75 g of 5‐ASA or 3 g POL per day.

### 2.5 Cell Culture

HIECs (3‐9007) and identification kits (3‐9012) were purchased from Chi Scientific Biotechnology. HIECs were grown in RPMI medium (0.25 U mL^−1^ insulin) with 10% FBS and 1% double‐antibody. The cells were incubated at 37 °C in 5% CO_2_. The medium was replaced every 48 h. Cells in the exponential growth phase were used for the experiments. Immunofluorescence staining and laser confocal microscopy were used to identify the expression of cytokeratin‐18.

Tg (T9033, Sigma) was used to establish an ERS model. Salubrinal (324895, Sigma), a selective inhibitor of the phosphatase complexes that dephosphorylate eIF2α, was used as a chemical blocker of ERS. HIECs were divided into six groups: ① control group (culture medium); ② Tg group (1 µM Tg, 12 h); ③ POL group (1 µM Tg, 12 h+1 mg mL^−1^ POL, 48 h); ④ 5‐ASA group (1 µM Tg, 12 h+ 20 mM 5‐ASA, 48 h)^[^ ^23^ ^]^; ⑤ Salubrinal group (1 µM Tg+10 µM Salubrinal, 12 h); ⑥ Salubrinal+POL group (1 µM Tg+10 µM Salubrinal, 12 h; 1 mg mL^−1^ POL, 48 h).

### 2.6 Cells Viability Assay

The viability of the HIECs was determined using an MTT assay. The effect of different concentrations of Tg and POL on cell viability was compared with that of untreated control cells, which were arbitrarily assigned a viability of 100%.

### 2.7 Histological Staining

Paraffin‐embedded colon samples were cut into 4 µm sections and stained with hematoxylin‐eosin (H&E). Samples were observed under an optical microscope (Olympus, Japan).

### 2.8 Transmission Electron Microscopy

Fresh colon tissues and cells were collected, fixed, dehydrated, rinsed, and embedded. The ultrathin sections (50‐60 nm) were double‐stained with 3% uranyl acetate and lead citrate and then observed with a transmission electron microscope (TEM).

### 2.9 Western Blot

The segments of mouse colon and cells were collected, and Western blot analyses were performed as previously described.^[^ ^24^ ^]^ The ECL reagents were used for detection. Image analysis was conducted using ImageJ software (Fiji, ImageJ).

For primary antibodies, anti‐eukaryotic initiation factor 2α (eIF2α)(#5324), anti‐phosphorylation of eukaryotic initiation factor 2α (p‐eIF2α) (#3398), anti‐autophagy related gene 7 (Atg7) (#8558), anti‐microtubule‐associated protein light chain 3II (LC3II)(#2775), anti‐actin (#4970), and secondary antibodies (Anti‐rabbit IgG, #7074 and Anti‐mouse IgG, #7076) were purchased from CST (MA, USA); anti‐activating transcription factor 4 (ATF4) (sc‐390063) and anti‐phosphorylation of protein kinase R‐like ER kinase (p‐PERK)(sc‐32577) were purchased from Santa Cruz (CA, USA); anti‐glucose‐regulated protein 78 (GRP78) (66574‐1‐lg) and anti‐protein kinase R‐like ER kinase (PERK)(24390‐1‐AP) were purchased from Protein Tech (IL, USA), anti‐Beclin1 (ab62557) was purchased from Abcam (Cambridge, UK).

### 2.10 Immunofluorescence Staining

Colon sections were incubated with anti‐LC3II (1:100, #2775, CST) and anti‐GRP78 (1:100, 66574‐1‐lg, Protein Tech) antibodies. Secondary antibodies (FITC‐conjugated AffiniPure goat anti‐mouse IgG, BA1101, TRITC‐conjugated AffiniPure goat anti‐rabbit IgG, BA1090, Boster, USA) were used in this study.

Cells were incubated with anti‐LC3II (1:500). Nuclei were stained with DAPI (1155MG010, Biofroxx, Germany). Images were obtained using a LEICA SP8 confocal microscope (LEICA, Germany). And the fluorescence intensity was quantified using ImageJ software (Fiji, ImageJ).

### 2.11 Enzyme‐Linked Immunosorbent Assay (ELISA)

NF‐κB p65, TNF‐α, and IL‐1β levels in the cell supernatant were measured using a human NF‐κB ELISA Kit (CSB‐12107h, Cusabio, China), TNF‐α ELISA Kit (DTA00D, Quantikine, USA), and IL‐1β ELISA Kit (DLB50, Quantikine, USA) according to the manufacturer's protocols.

### 2.12 Statistical Analysis

Each experiment was repeated at least three times with three replications per experiment. All data are expressed as the mean ± SEM. Statistical analysis was performed using GraphPad Prism (version 8.3, GraphPad). Data with a normal distribution and homogeneity between groups were compared using one‐way ANOVA; nonnormally distributed data were analyzed with nonparametric tests. Tukey's multiple comparisons test was used to compare between groups. A *p*‐value of <0.05 was considered statistically significant.

## 3 Results

### 3.1 Phytochemical Identification of POL

**Figure 1:**
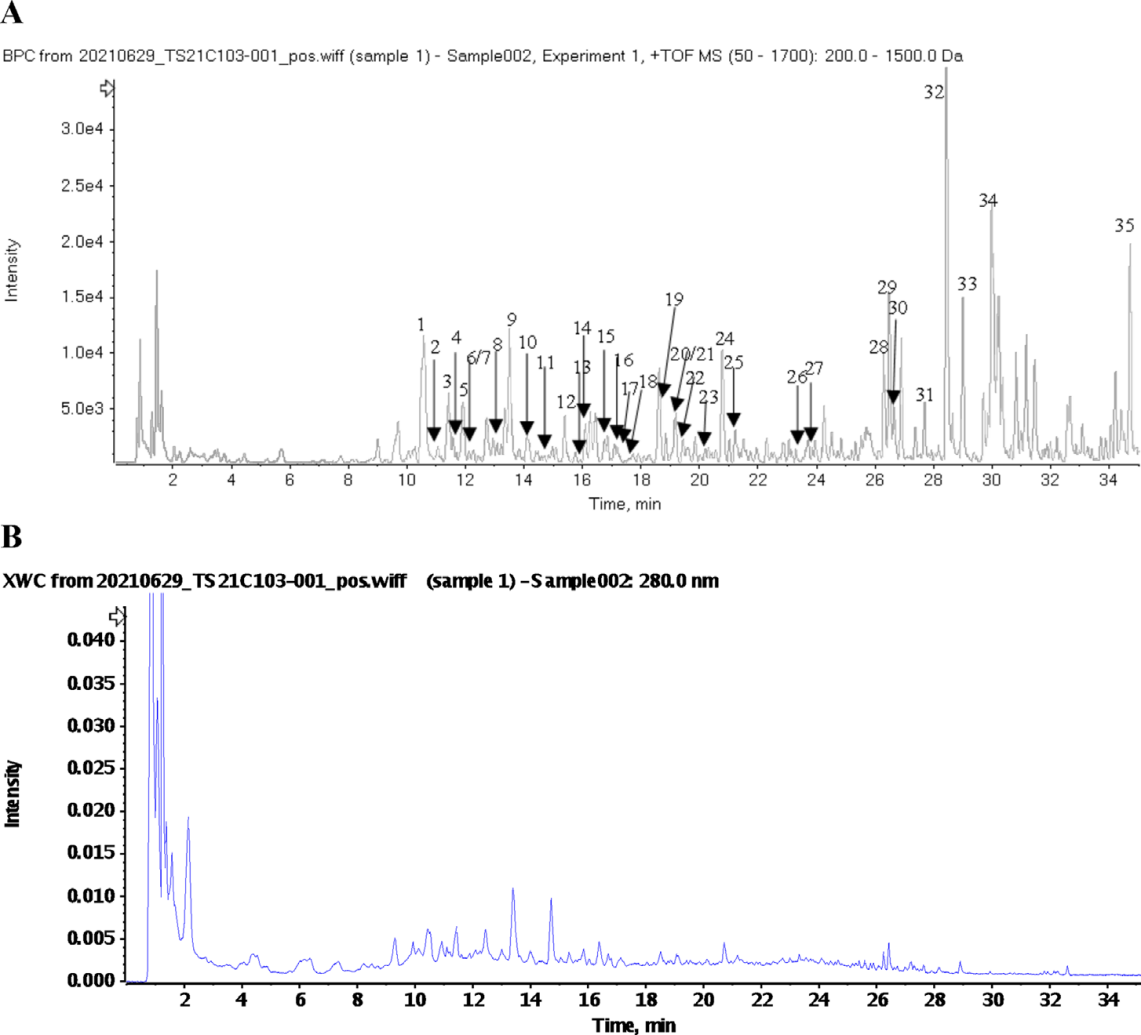
UPLC‐Q‐TOF/MS analysis of POL Extract. A) Base peak ion (BPI) chromatogram of POL extract in positive ion mode determined by UPLC‐HRMS analysis; B) HPLC‐UV chromatogram of POL extract (280 nm).

The extract of POL contains various active components, and UPLC‐Q‐TOF/MS is used to analyze the chemical components of TCM due to its high resolution, sensitivity, and accuracy.^[^ ^25^ ^]^ Therefore, we performed constituent identification using UPLC‐Q‐TOF/MS. Thirty‐five compounds were identified from POL according to multistage mass spectrometry information and the high‐resolution mass spectrometry database of natural products (**Table S2**, Supporting Information). The UPLC‐Q‐TOF/MS chromatographic profile is shown in **Figure** **1**.

### 3.2 POL Improves the Symptoms and Colonic Inflammation in PAC IL‐10^–/–^ Mice

**Figure 2:**
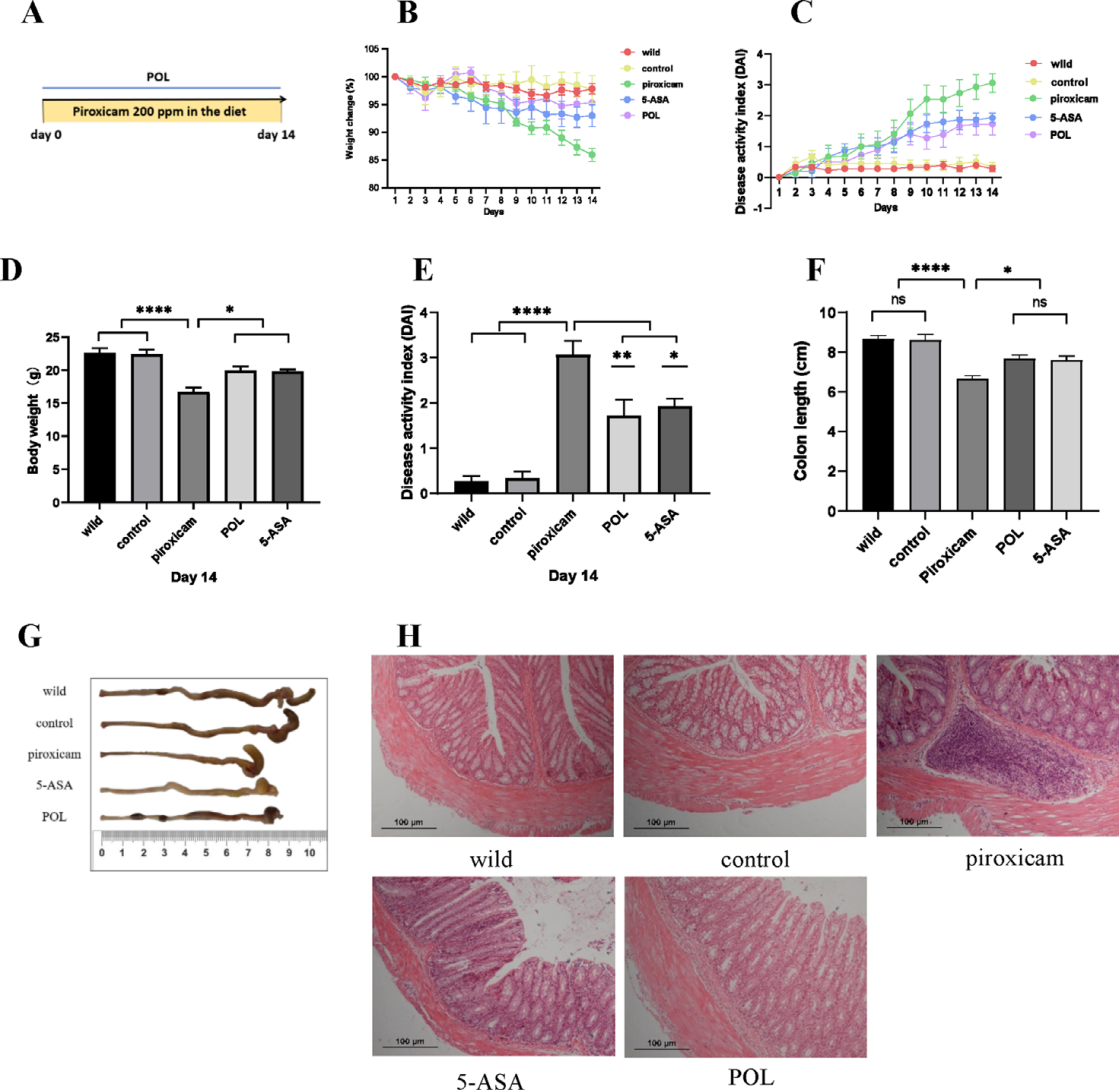
POL improves the symptoms and colonic inflammation in PAC IL‐10^–/–^ mice. A) The flow chart of mouse experiment. A murine model of colitis induced by piroxicam in IL‐10^–/–^ mice. Normal saline, 5‐ASA (100 mg kg^−1^ d^−1^) or POL (400 mg kg^−1^ d^−1^) was given intragastrically daily to the mice in the different groups. B and C) The body weight and DAI scores were recorded daily (*n* = 5/6). D and E) The statistical graph of body weight and DAI scores in Day 14 (*n* = 5/6). F and G) The colon length and statistical graph (*n* = 5/6). *p < 0.05, **p < 0.01, ****p < 0.0001. H) H&E staining of colons (×100). 5‐ASA, 5‐aminosalicylic acid; DAI, disease activity index; H&E, hematoxylin and eosin; POL: *Portulaca oleracea L*.

To evaluate the therapeutic effect of POL on IBD, a PAC IL‐10^–/–^ mouse model was established (**Figure** **2**A). One mouse in each of the piroxicam and 5‐ASA groups died before the end of the experiment. Higher body weight loss (Figure 2B,D) and DAI scores (Figure 2C,E) were observed in the piroxicam group than in the control and wild‐type groups. In addition, the length of the colon was shorter in the piroxicam group (Figure 2F,G). Treatment with 5‐ASA and POL improved these symptoms in PAC IL‐10^–/–^ mice.

Moreover, H&E staining showed clear and intact colonic epithelial structures, complete and continuous mucosae, healthy crypt structure, abundant goblet cells, and neatly arranged glands, without inflammatory cell infiltration in the wild‐type and control groups. In contrast, the piroxicam group showed severe epithelial injury, irregular glands, inflammatory cell infiltration, and ulcer formation. Treatment with 5‐ASA and POL alleviated the damage to colonic tissue. The intestinal villi were relatively intact. Inflammatory cell infiltration, crypt abscess, and ulcer formation were significantly ameliorated (Figure 2H). These data suggested that 5‐ASA and POL may promote the healing effect of colitis.

### 3.3 POL Inhibits ERS and Regulates Autophagy in PAC IL‐10^–/–^ Mice

**Figure 3:**
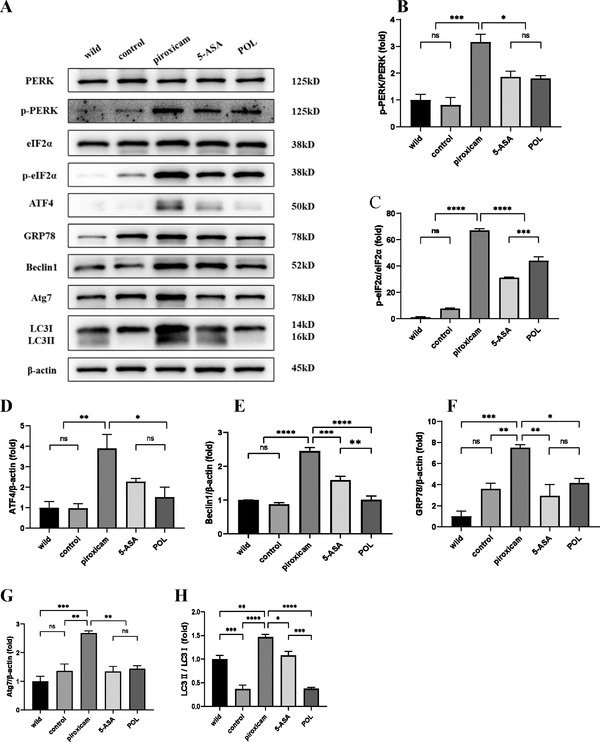
POL inhibits ERS and regulates autophagy in PAC IL‐10^–/–^ mice. A) The expression levels of PERK, p‐PERK, eIF2α, p‐eIF2α, ATF4, GRP78, Beclin1, Atg7, LC3I, LC3II, and β‐actin were assessed by western blot. B–H) Relative expression of p‐PERK, p‐eIF2α, ATF4, Beclin1, GRP78, Atg7, and LC3II in the wild‐type, control, piroxicam, 5‐ASA, and POL groups. Data are presented as the mean ± SEM. *p < 0.05, **p < 0.01, ***p < 0.001, ****p < 0.0001. 5‐ASA, 5‐aminosalicylic acid; ATF4, activating transcription factor 4; Atg7, autophagy related gene 7; eIF2α, eukaryotic initiation factor 2α; GRP78, glucose‐regulated protein 78; LC3II, microtuble‐associated protein light chain 3II; POL, *Portulaca oleracea L*.; PERK, protein kinase R‐like ER kinase; p‐PERK, phosphorylation of protein kinase R‐like ER kinase; p‐eIF2α, phosphorylation of eukaryotic initiation factor 2α.

To investigate whether ERS and autophagy were attenuated by POL‐mediated protection in PAC IL‐10^–/–^ mice, ERS and autophagy‐related molecules in colon lysates were measured by Western blot. ERS‐related proteins, including p‐PERK, p‐eIF2α, GRP78, and ATF4, as well as autophagy‐related proteins, including Beclin1, Atg7, and LC3II, were significantly increased in the piroxicam group compared to the wild‐type and control groups. This process was reversed by 5‐ASA and POL treatment (**Figure** **3**A‐H), suggesting that colitis remission induced by POL was associated with downregulation of the ERS‐autophagy pathway.

**Figure 4:**
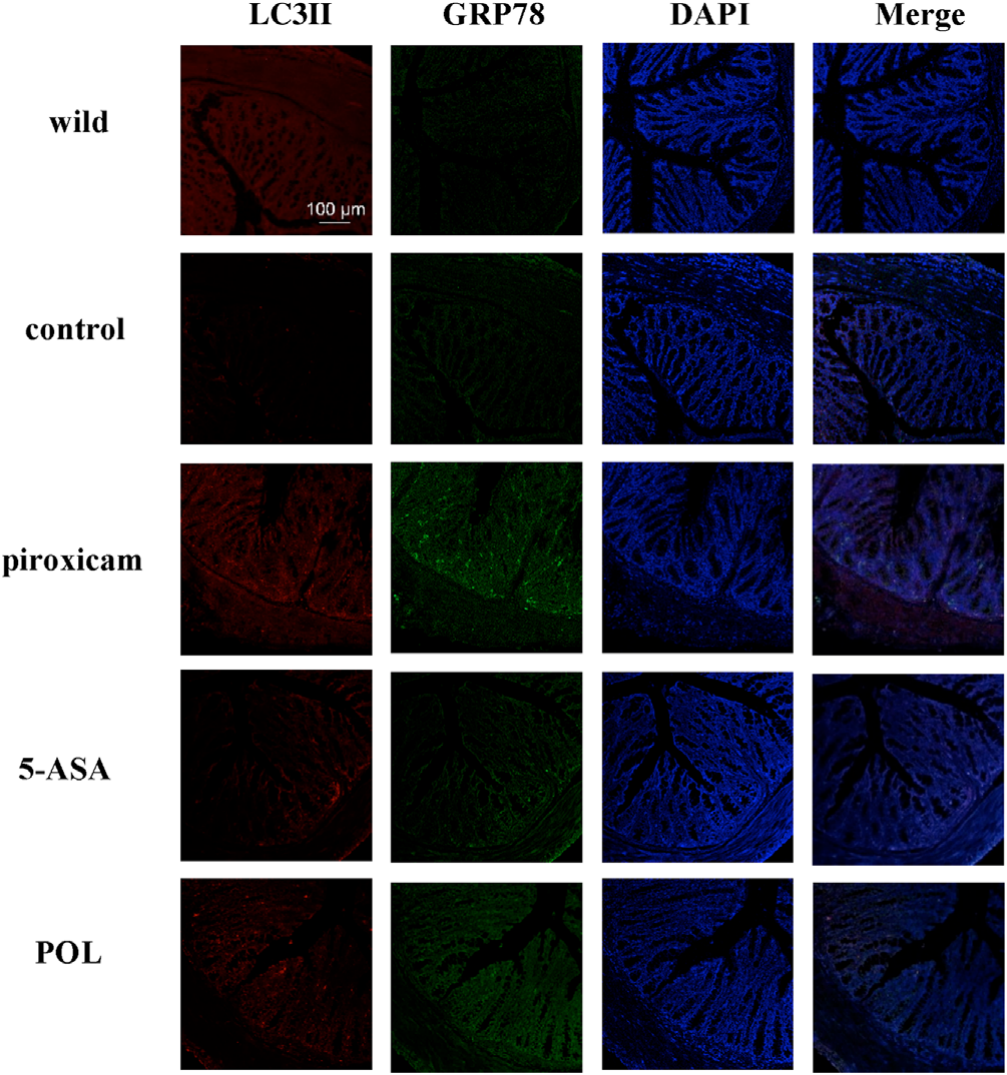
Immunofluorescence staining for LC3II (red) and GRP78 (green) in mouse colons (×100). 5‐ASA, 5‐aminosalicylic acid; GRP78, glucose‐regulated protein 78; LC3II, microtuble‐associated protein light chain 3II; POL, *Portulaca oleracea L*.

Next, we evaluated LC3II and GRP78 expression in the colon by immunofluorescence. Our results revealed high expression of LC3II and GRP78 in piroxicam‐induced IL‐10^–/‐^ mice; these levels were significantly reduced in the POL and 5‐ASA groups (**Figure** **4**).

**Figure 5:**
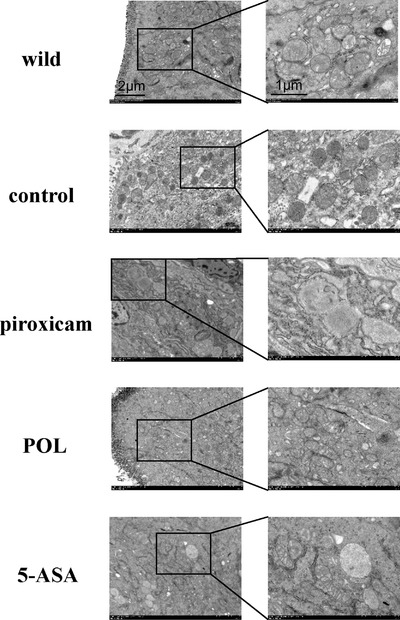
The ultrastructure of the ER and autophagosomes in IECs were observed by TEM (×6000, ×15 000). 5‐ASA, 5‐aminosalicylic acid; ER, endoplasmic reticulum; IECs, intestinal epithelial cells; POL, *Portulaca oleracea L*.; TEM, transmission electron microscope.

The ultrastructure of the colon was evaluated by TEM. The clear, fascicular, and undilated ER, healthy mitochondrial, and few autophagosome in the cytoplasm in the wild‐type group. In control group, the mitochondrial were slightly swollen. In contrast, in the piroxicam group, the number of rough ER increased, and the ER lumen was expanded, and they were vacuolar and sometimes fused into clusters. The number of autophagosome significantly increased. In the POL group, the number of rough ERs was reduced compared with that in the piroxicam group, and the ER was slightly dilated. The morphology tended to be membrane‐like, and the number of autophagosome decreased. In the 5‐ASA group, the ER was slightly dilated, and the morphology basically returned to normal, showing a cluster‐like distribution. The mitochondrial cristae were clear and regular, and the number of autophagosome decreased compared to the piroxicam group (**Figure** **5**).

### 3.4 HIEC Morphology and the Effects of Tg and POL on Cell Viability In Vitro

**Figure 6:**
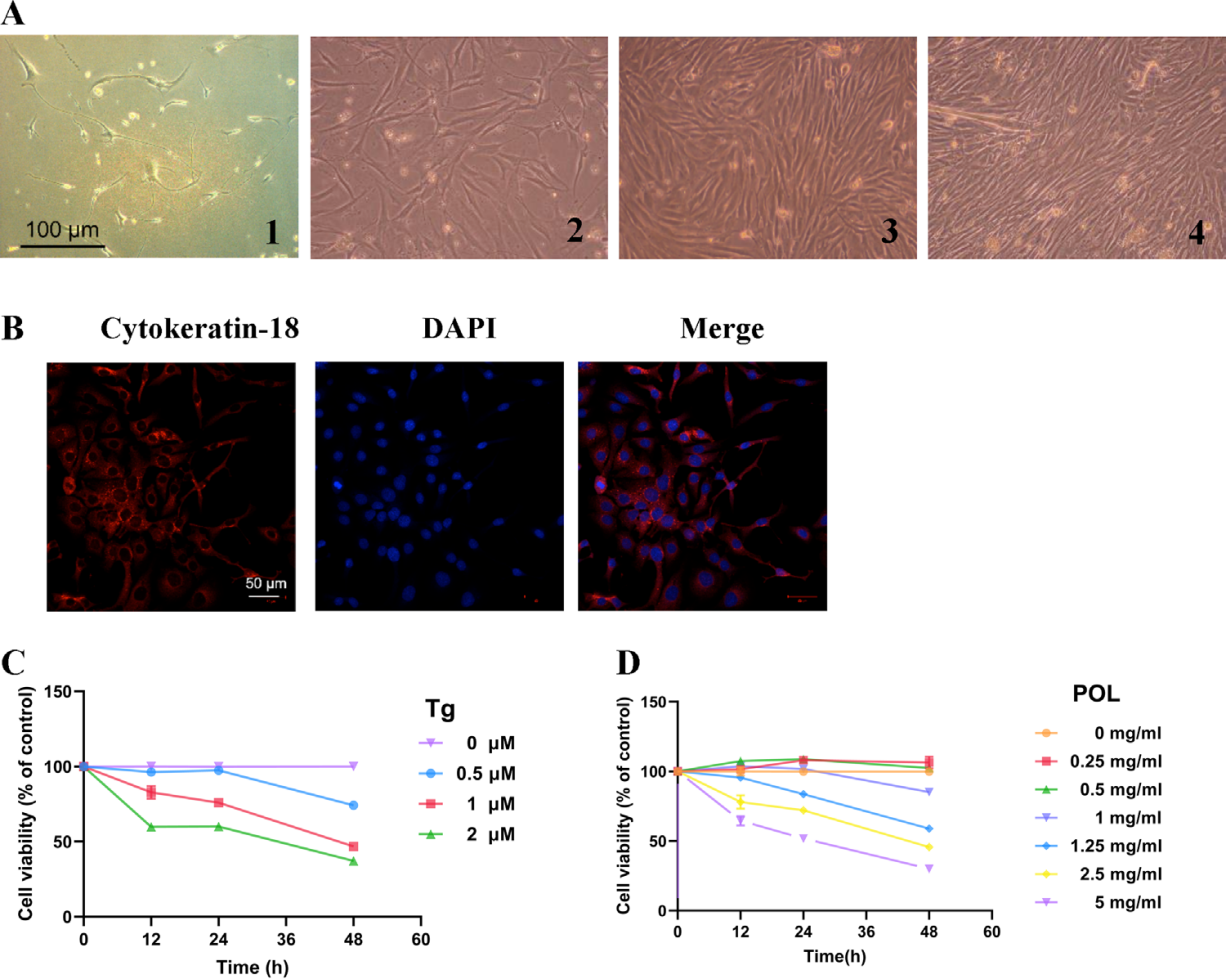
Effects of Tg and POL on cell viability. A) Morphology of HIECs under an optical microscope. HIECs were cultured A1) for 2 d, A2) for 6 d, A3) for 12 d, and A4) for 13 d from left to right (×100). B) Morphology of HIECs under a confocal microscope (×200), cytokeratin‐18 (red), and DAPI (blue). C and D) HIECs were treated with various concentrations of Tg or POL for 12, 24, and 48 h. Cell viability was measured with MTT assay. HIECs, human intestinal epithelial cells; POL, *Portulaca oleracea L*.; Tg, thapsigargin.

HIECs adhered to the wall within 24–48 h. The adherent HIECs grew in monolayers with different shapes, most of which were polygonal, such as triangular or polygonal, and fusiform. There were many protrusions on the cell surface, the cytoplasm was full, and the nucleus was round or oval, which was consistent with the characteristics of epithelial cells. The cells formed a cell community in 5–6 days and were confluence in 9–11 days. The cells were closely arranged and resembled paving stones (**Figure** **6**A). Immunofluorescence laser confocal microscopy showed that cytokeratin‐18 expression was positive, indicating that the cells were human colonic epithelial cells (Figure 6B).

Next, we used the MTT assay to analyze cell viability after drug treatment. The concentration >1 µM Tg significantly decreased cell activity over time, indicating that a high concentration of Tg may be toxic to the cells (the average cell activity was 82.83% when treated with 1 µmol L^‐1^ Tg for 12 h) (Figure 6C).

The concentrations of 1, 1.25, 2.5, and 5 mg mL^−1^ POL decreased the cell activity over time, indicating that POL has a certain toxic effect. The average cell activity was 85.21% when treated with 1 mg mL^‐1^ for 48 h (Figure 6D). Therefore, 1 µmol L^‐1^ Tg and 1 mg mL^‐1^ POL were selected for further experiments.

### 3.5 POL Inhibits the Production of Proinflammatory Mediators in Tg‐Induced HIECs

**Figure 7:**
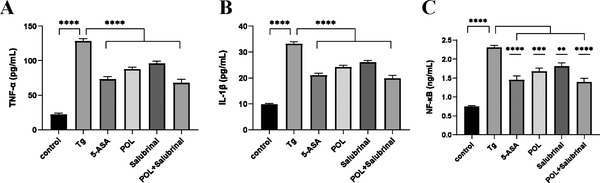
POL inhibits the production of proinflammatory mediators in Tg‐induced HIECs. A) TNF‐α, B) IL‐1β, and C) NF‐κB in colon supernatant were determined by ELISA. Data are presented as the mean ± SEM. **p < 0.01, ***p < 0.001, ****p < 0.0001. 5‐ASA, 5‐aminosalicylic acid; HIECs, human intestinal epithelial cells; IL‐1β, interleukin‐1β; NF‐kB, nuclear factor kappa‐B; POL, *Portulaca oleracea L*.; Tg, thapsigargin; TNF‐α, tumor necrosis factor–α.

Tg is most commonly used to perturb ER homeostasis. In this study, we used Tg to induce an ERS cell model. To determine whether POL exhibits anti‐inflammatory action in HIECs, TNF‐α, IL‐1β, and NF‐κB were measured by ELISA. The expression of TNF‐α, IL‐1β, and NF‐κB increased significantly in Tg‐induced HIECs, while POL and 5‐ASA reversed this effect (**Figure** **7**A‐C).

### 3.6 POL Alleviated Autophagy in Tg‐Induced ERS in HIECs

**Figure 8:**
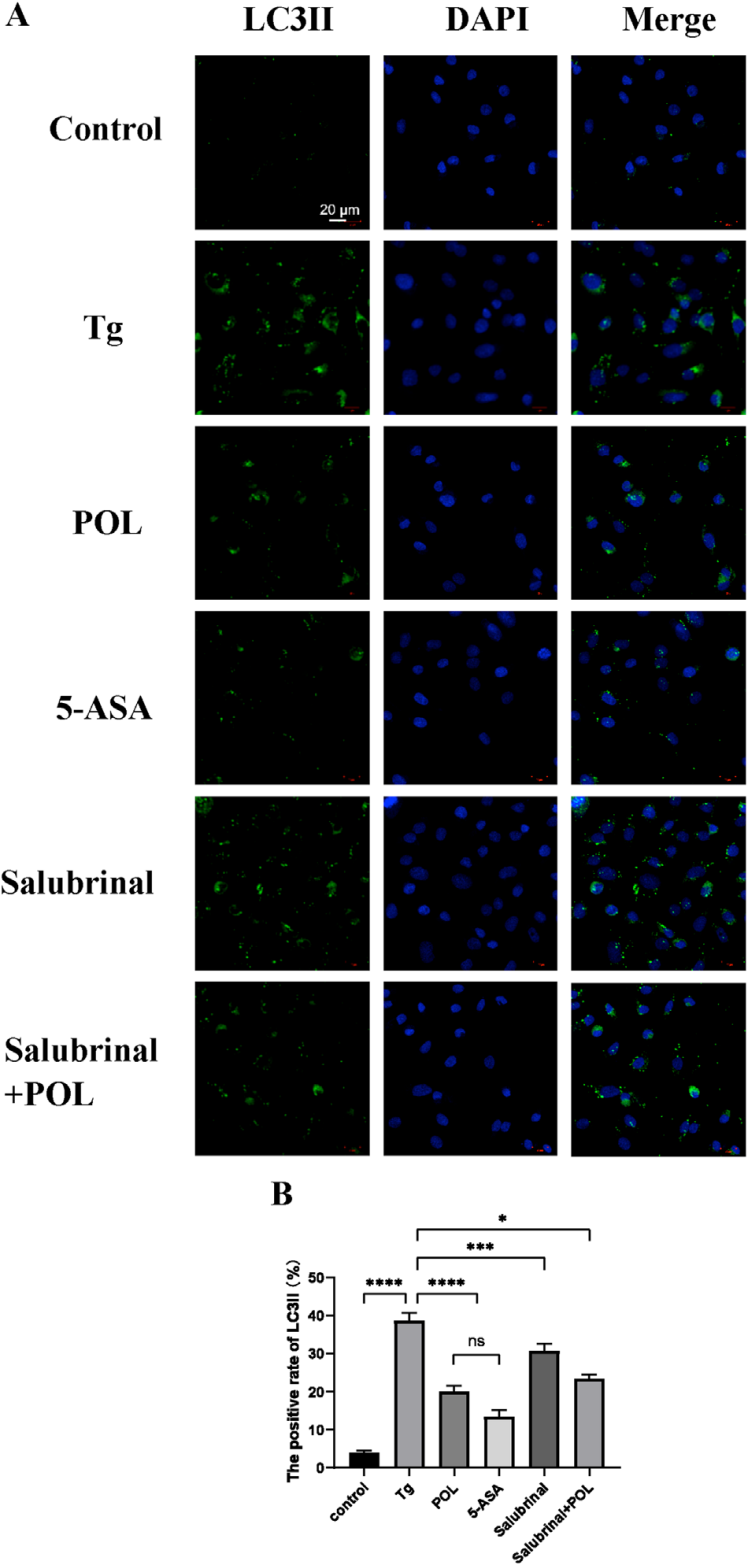
A) Immunofluorescence staining for LC3II in HIECs (×630), LC3II (green), and DAPI (blue). B) The positive rate of LC3II in HIECs (%). 5‐ASA, 5‐aminosalicylic acid; HIECs, human intestinal epithelial cells; POL, *Portulaca oleracea L*.; Tg, thapsigargin. *p < 0.05, ***p < 0.001, ****p < 0.0001.

**Figure 9:**
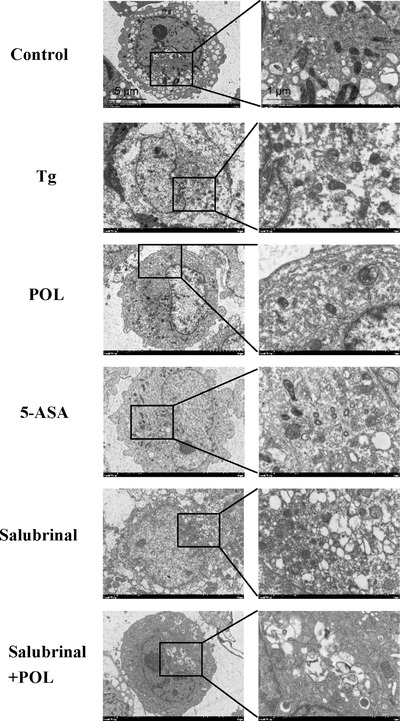
The ultrastructure of mitochondrial and autophagosomes in HIECs (×3000, ×10 000). 5‐ASA, 5‐aminosalicylic acid; HIECs, human intestinal epithelial cells; POL, *Portulaca oleracea L*.; Tg, thapsigargin.

To clarify the effect of Tg‐induced ERS on autophagy and POL intervention in HIECs, TEM was used to observe the autophagosomes of IECs, and immunofluorescence staining was used to detect autophagy. The green fluorescent label LC3II is located on the autophagosome membrane. The fluorescence intensity of LC3II was significantly enhanced in the Tg group compared to the control group and reduced to varying extents in different treatment groups in HIECs (**Figure** **8**A,B). We also observed a significantly larger number of autophagosomes in Tg group compared to the control group by TEM. Mitochondrial and ribosomes were surrounded by autophagosomes. The numbers of autophagosomes decreased after treatment with POL or 5‐ASA. The submicroscopic structure of HIECs was normalized (**Figure** **9**).

### 3.7 POL Reduces the Expression of ERS and Autophagy‐Related Proteins in Tg‐Induced HIECs

**Figure 10:**
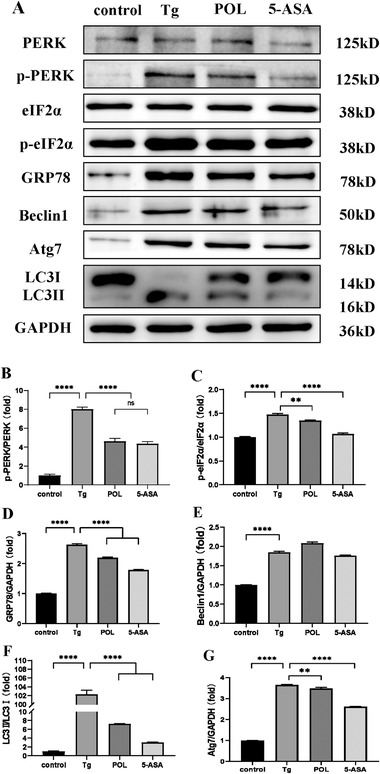
POL reduces the expression of ERS and autophagy‐related proteins in Tg‐induced HIECs. A) The expression levels of PERK, p‐PERK, eIF2α, p‐eIF2α, GRP78, Beclin1, Atg7, LC3I, LC3II, and GAPDH were assessed by western blot. B–G) Relative expression of p‐PERK, p‐eIF2α, GRP78, Beclin1, LC3II, and Atg7 in the control, Tg, POL, and 5‐ASA groups. Data are presented as the mean ± SEM. **p < 0.01, ****p < 0.0001. 5‐ASA, 5‐aminosalicylic acid; Atg7, autophagy related gene 7; eIF2α, eukaryotic initiation factor 2α; GRP78, glucose‐regulated protein 78; LC3II, microtubule associated protein light chain 3II; p‐eIF2α, phosphorylation of eukaryotic initiation factor 2α; PERK, protein kinase R‐like ER kinase; p‐PERK, phosphorylation of protein kinase R‐like ER kinase; POL, *Portulaca oleracea L*.; Tg, thapsigargin.

Activation of the PERK signaling pathway occurs in the early stage of ERS. We assessed PERK, p‐PERK, eIF2α, and p‐eIF2α expression in Tg‐induced ERS in HIECs by Western blot. The expression of p‐PERK and p‐eIF2α significantly increased in Tg‐treated HIECs and decreased in the POL and 5‐ASA groups. The protein GRP78, a molecular chaperone, belongs to the HSP70 family and is involved in the folding and trafficking of secretory and transmembrane proteins, consistent with the increased expression of p‐PERK and p‐eIF2α. Simultaneously, autophagy‐related proteins, including Beclin1, Atg7, and LC3II/I, significantly increased after treatment with Tg compared with the control group. POL and 5‐ASA reversed the efficacy of Tg (**Figure** **10**A‐G).

**Figure 11:**
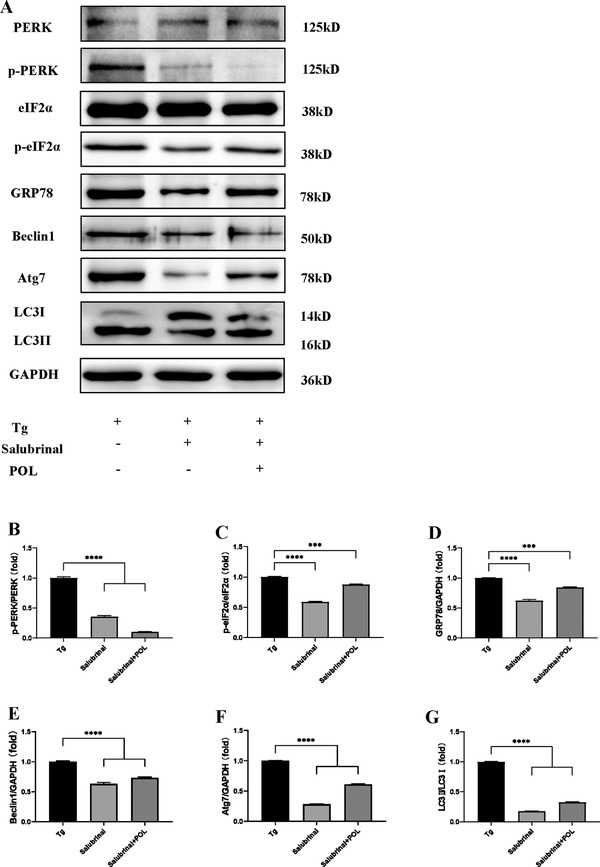
POL reduces the expression of ERS and autophagy‐related proteins in Tg‐induced HIECs. A) The expression levels of PERK, p‐PERK, eIF2α, p‐eIF2α, GRP78, Beclin1, Atg7, LC3I, LC3II, and GAPDH were assessed by western blot. B‐G) Relative expression of p‐PERK, p‐eIF2α, GRP78, Beclin1, Atg7, and LC3II. Data are presented as the mean ± SEM. ***p < 0.001, ****p < 0.0001. 5‐ASA, 5‐aminosalicylic acid; Atg7, autophagy related gene 7; eIF2α, eukaryotic initiation factor 2α; GRP78, glucose‐regulated protein 78; LC3II, microtubule associated protein light chain 3II; PERK, protein kinase R‐like ER kinase; p‐PERK, phosphorylation of protein kinase R‐like ER kinase; p‐eIF2α, phosphorylation of eukaryotic initiation factor 2α; POL, *Portulaca oleracea L*.; Tg, thapsigargin.

To verify the role of the PERK‐eIF2α/Beclin1‐LC3II pathway in ERS‐induced autophagy in vitro, we pretreated HIECs with salubrinal, an eIF2α Inhibitor, and then stimulated them with Tg. GRP78, Beclin1, and LC3II protein expression decreased after salubrinal pretreatment, suggesting that the PERK‐eIF2α/Beclin1‐LC3II pathway was related to ERS‐induced autophagy and that ERS inhibition could reduce the autophagy response. The expression levels of ERS‐autophagy‐related proteins, such as p‐PERK, p‐eIF2α, GRP78, Beclin1, ATG7, and LC3II, all decreased after POL intervention, suggesting that POL might alleviate colitis through the PERK‐eIF2α/Beclin1‐LC3II pathway, inhibiting the phosphorylation of the upstream PERK‐eIF2α pathway and subsequently inhibiting the expression of the autophagy marker proteins Beclin1 and LC3II (**Figure** **11**A‐G).

## 4 Discussion

IBD is a chronic disease caused by the interaction of environmental, immune, genetic and other factors.^[^ ^26^ ^]^ Identifying the common mechanisms underlying pathogenesis and potential therapeutic targets is urgently needed. ER is an important organelle in eukaryotic cells, playing an important role in protein synthesis, processing, transport, lipid synthesis, and the storage and release of Ca^2+^. ERS is characterized by incorrect folding and aggregation of unfolded proteins in the endoplasmic reticulum lumen and a disruption of calcium homeostasis and lipid metabolism caused by hypoxia, oxidative stress, drugs, toxins, and nutrient deprivation.^[^ ^9^, ^27^ ^]^ Numerous studies have shown that ERS is involved in the pathology of IBD, and inhibiting excessive ERS can attenuate mouse colitis.^[^ ^28^, ^29^ ^]^

Disrupting autophagy triggers inflammation, thus increasing the risk of intestinal diseases.^[^ ^30^, ^31^ ^]^ There is growing evidence suggesting that ERS induces autophagy when unfolded proteins are overaccumulated, and ERS and autophagy are closely related and may influence each other.^[^ ^32^, ^33^, ^34^ ^]^ Some researchers suggest that autophagy‐dependent apoptosis or autophagy‐dependent survival induced by ERS depends on the cell type and stimulus severity.^[^ ^35^ ^]^ Moderate autophagy can inhibit ERS by clearing intracellular unfolded proteins and damaged organelles, to help maintain cellular homeostasis, while excessive autophagy may result in cell death, which is designated type II programmed cell death (autophagic cell death).^[^ ^36^ ^]^ Drugs, such as Tg and tunicamycin, that directly disrupt the environment and function of the ER can induce autophagy.^[^ ^37^, ^38^ ^]^

A variety of autophagy‐related factors are involved in ERS‐induced autophagy in IBD. PERK is one of the three canonical ER transmembrane protein sensors of the UPR. Upon ER stress, the chaperone GRP78 is released from PERK, which then undergoes oligomerization and trans‐autophosphorylation.^[^ ^39^ ^]^ p‐PERK specifically induces phosphorylation of eIF2α on serine 51 and attenuates protein translation, decreasing the ER protein‐folding load. Simultaneously, p‐eIF2α results in upregulation of ATF4, which induces the expression of various genes to maintain ER homeostasis.^[^ ^40^, ^41^, ^42^ ^]^ Beclin1 is a mammalian ortholog of yeast autophagy‐related gene 6 (Atg6). Upon starvation or other stimuli, Beclin1 is upregulated/activated, leading to the initiation of membrane/organelle isolation and phagophore formation.^[^ ^43^ ^]^ Further membrane expansion/fusion and autophagosome formation require microtubule‐associated protein 1A/1B light chain 3B (LC3).^[^ ^44^ ^]^ Therefore, the PERK‐eIF2α/Beclin1‐LC3II pathway may be involved in the pathogenesis of IBD and may become a new therapeutic target for intervention.

POL, a homolog of certain medicines and food, is rich in polysaccharides, flavonoids, alkaloids, organic acids, multiple vitamins and trace elements, and other chemical components. POL has been used as a traditional medicine for alleviating a wide spectrum of diseases not only in China but also in Europe. Dioscorides, the father of pharmacology, mentioned the medicinal properties of this plant in his pharmacology book De Materia Medica.^[^ ^11^ ^]^ Recent studies have revealed that POL suppresses lung inflammation and relieves ethanol‐induced liver cells and zymosan‐induced joint inflammation by reducing IL‐β, IL‐6, and TNF‐α, as well as by increasing IL‐10 levels.^[^ ^13^, ^45^, ^46^ ^]^ Based on network pharmacology, POL is considered a multitarget agent whose pharmacological response is associated with the modulation of multiple targets/pathways, which may be advantageous against complex diseases such as IBD.^[^ ^47^, ^48^ ^]^ Previous experimental studies have shown that POL inhibits the oxidative stress response, reduces the expression of proinflammatory cytokines (TNF‐α, IL‐1β, and IL‐6),^[^ ^12^ ^]^ diminishes the symptoms of colitis and improves the colon histopathological structure of DSS‐induced colitis mice, similar to treatment with 5‐ASA.^[^ ^13^, ^45^ ^]^ In our study, POL decreased the expression of TNF‐α, IL‐1β, and NF‐κB and alleviated colitis, consistent with previous studies. POL suppressed the increased expression of p‐PERK and its downstream targets. Autophagy‐related proteins, such as Beclin1 and LC3II, were decreased after POL treatment. The results potentially strengthen the therapeutic rationale of POL for IBD by blocking the PERK‐eIF2α/Beclin1‐LC3II pathway. Nevertheless, there is a limitation in this study. We did not compare the effects of POL between sexes because of the limited number of IL‐10^–/–^ mice. Further studies should be conducted to confirm the efficacy of POL in humans.

## 5 Conclusion

**Figure 12:**
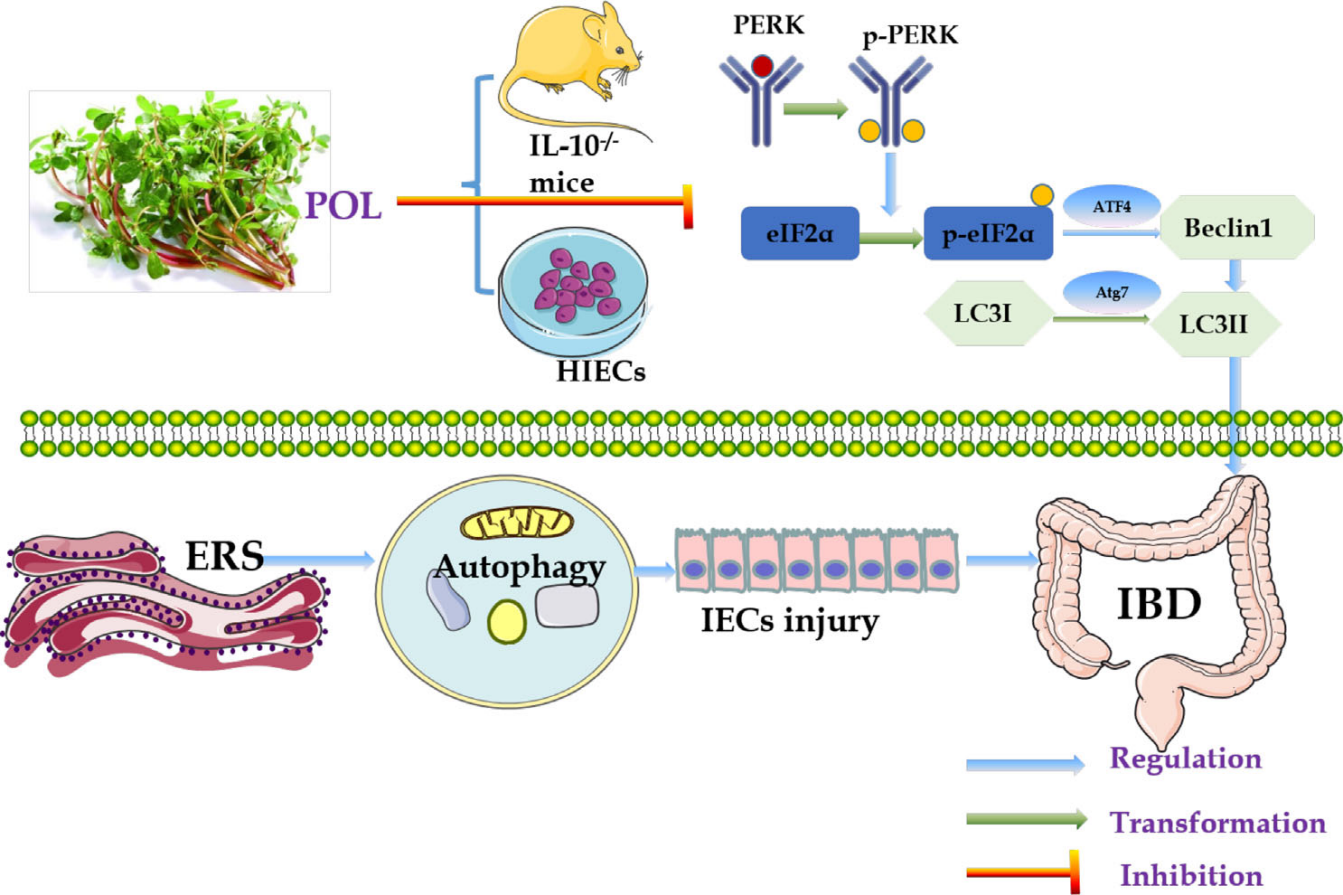
Schematic diagram of the therapeutic mechanism through which POL alleviates ERS‐autophagy damage during the development of IBD. ATF4, activating transcription factor 4; Atg7, autophagy‐related gene 7; ERS, endoplasmic reticulum stress; eIF2α, eukaryotic initiation factor 2α; HIECs, human intestinal epithelial cells; IBD, inflammatory bowel disease; IECs, human intestinal epithelial cells; IL‐10, interleukin‐10; LC3I, microtubule‐associated protein light chain 3I; LC3II, microtubule‐associated protein light chain 3II; PERK, protein kinase R‐like ER kinase; p‐eIF2α, phosphorylation of eukaryotic initiation factor 2α; POL, *Portulaca oleracea L*.; p‐PERK, phosphorylation of protein kinase R‐like ER kinase.

To investigate the role of ERS‐induced autophagy in IBD and the protective and anti‐inflammatory mechanism of POL extract, we established PAC IL‐10^–/–^ model and Tg‐stimulated ERS model of HIECs. It was found that POL exerts a therapeutic effect by reducing the expression of proinflammatory factors such as IL‐1β, TNF‐α, and NF‐κB and repairing intestinal mucosal damage. Moreover, the Western Blot and Immunofluorescence showed POL extracts reduced the damage of IECs by regulating the PERK‐eIF2α/Beclin1‐LC3II pathway (**Figure** **12**) and the use of salubrinal, an ER‐stress inhibitor confirms that. This study further confirmed the anti‐inflammatory mechanism and protective effect of POL extract and may offer new ideas for effective treatment of IBD.

## Conflict of Interest

The authors declare no conflict of interest.

## Author Contributions

Z.Z. and D.Q. contributed equally to this work; Y.D. and Z.T. designed the experiments; Y.Z., Q.C., Y.C., Y.T., R.Y., Y.C., and L.Z. performed the experiments; Z.Z., D.Q., and Y.D. developed and wrote the manuscript; Y.D. and Z.T. revised and edited the manuscript.

## Supporting information

Supporting Information

## Data Availability

The datasets generated during and/or analyzed during the current study will be available upon request from principle investigator. The shared data will only be allowed to be used by the applicant for scientific studies. No commercial activities are allowed.
