# Supplementary material for: Portulaca oleracea L. Extract Ameliorates Intestinal Inflammation by Regulating Endoplasmic Reticulum Stress and Autophagy
Source: Mol Nutr Food Res. 2022 Jan 9;66(5):2100791. doi: 10.1002/mnfr.202100791 (PMC9286603; doi:10.1002/mnfr.202100791)
Supplement: Supplementary file 1 — Supporting Information [file MNFR-66-0-s001.docx]

**Table S1. The gradient elution program of the mobile phase**

| Time (min) | Flow Rate (mL/min) | B% |
| --- | --- | --- |
| 0 | 0.3 | 5 |
| 5 | 0.3 | 5 |
| 10 | 0.3 | 15 |
| 15 | 0.3 | 20 |
| 20 | 0.3 | 30 |
| 25 | 0.3 | 50 |
| 30 | 0.3 | 70 |
| 35 | 0.3 | 95 |

**Table S2. Identification results of main components of POL Extract.**

| NO | Retention time（min） | Adducts | Measured (M/Z) | Expected (M/Z) | Error(ppm) | Formula | Molecular  weight | Phytochemical name | MS/MS spectra |
| --- | --- | --- | --- | --- | --- | --- | --- | --- | --- |
| 1 | 10.59 | [M+H]^+^ | 220.0965 | 220.0968 | -1.5 | C_12_H_13_NO_3_ | 219.09 | Oleracein E | 220.0976;137.0603;119.0491;91.0542 |
| 2 | 11.16 | [M+H]^+^ | 828.2579 | 828.2557 | 2.7 | C_36_H_45_NO_21_ | 827.25 | Oleracein P | 828.2639;666.2061;504.1490;342.0945;147.0449 |
| 3 | 11.42 | [M+H]^+^ | 256.1337 | 256.1332 | 1.9 | C_16_H_17_NO_2_ | 255.31 | 1,​2,​3,​4-​tetrahydro-​1-​(phenylmethyl)​-6,​7-​isoquinolinediol | 256.1323;239.1063;178.0757;161.0581;91.0541 |
| 4 | 11.68 | [M+H]^+^ | 858.2679 | 858.2662 | 1.9 | C_37_H_47_NO_22_ | 857.26 | Oleracein Q | 858.2715;696.2135;534.1572;372.1037;177.0542 |
| 5 | 11.96 | [M+H]^+^ | 666.2034 | 666.2029 | 0.8 | C_30_H_35_NO_16_ | 665.20 | Oleracein H | 666.2026;504.1511;342.1002;147.0444 |
| 6 | 12.26 | [M+H]^+^ | 211.1443 | 211.1441 | 0.9 | C_11_H_18_N_2_O_2_ | 210.14 | Cyclo(Ile-Pro) | 211.1447;183.1487;154.0724;138.1271 |
| 7 | 12.49 | [M+H]^+^ | 696.2151 | 696.2134 | 2.4 | C_31_H_37_NO_17_ | 695.21 | Oleracein I | 696.2206;534.1649;372.1117;177.0549;145.0278 |
| 8 | 13.09 | [M+H]^+^ | 211.1444 | 211.1441 | 1.4 | C_11_H_18_N_2_O_2_ | 210.14 | Cyclo(Leu-Pro) | 211.1451;183.1510;138.1274;114.0907 |
| 9 | 13.62 | [M+H]^+^ | 666.2011 | 666.2029 | -2.6 | C_30_H_35_NO_16_ | 665.20 | Oleracein C | 504.1530;342.0974;147.0433 |
| 10 | 14.17 | [M+H]^+^ | 696.2118 | 696.2134 | -2.3 | C_31_H_37_NO_17_ | 695.21 | Oleracein D | 696.2161;534.1581;372.1068;177.0540;145.0292 |
| 11 | 14.85 | [M+H]^+^ | 245.1272 | 245.1285 | -5.1 | C_14_H_16_N_2_O_2_ | 244.12 | Cyclo(Phe-Pro) | 245.1267;217.1322;154.0734;120.0800 |
| 12 | 15.41 | [M+H]^+^ | 504.1503 | 504.15 | 0.5 | C_24_H_25_NO_11_ | 503.14 | Oleracein A | 342.0955;147.0436;119.0500 |
| 13 | 15.94 | [M+H]^+^ | 1004.3057 | 1004.303 | 2.7 | C_46_H_53_NO_24_ | 1003.30 | Oleracein R | 1004.2978;504.1494;501.1585;342.0951;177.0531 |
| 14 | 16.11 | [M+H]^+^ | 534.1609 | 534.1606 | 0.6 | C_25_H_27_NO_12_ | 533.15 | Oleracein B | 534.1633;372.1063;177.0539;145.0283 |
| 15 | 16.78 | [M+H]^+^ | 842.2495 | 842.2502 | -0.8 | C_40_H_43_NO_19_ | 841.24 | Oleracein N | 504.1487;342.0960;339.1069;177.0538 |
| 16 | 17.19 | [M+H]^+^ | 872.2611 | 872.2608 | 0.4 | C_41_H_45_NO_20_ | 871.25 | Oleracein O | 872.2588;534.1599;339.1065;177.0541 |
| 17 | 17.32 | [M+H]^+^ | 828.2341 | 828.2346 | -0.5 | C_39_H_41_NO_19_ | 827.23 | Oleracein K | 828.2309;504.1540;342.0971;147.0444 |
| 18 | 17.45 | [M+H]^+^ | 858.2407 | 858.2451 | -5.2 | C_40_H_43_NO_20_ | 857.24 | Oleracein L | 858.2404;534.1627;372.1092;177.0538 |
| 19 | 18.75 | [M+H]^+^ | 284.1276 | 284.1281 | -1.8 | C_17_H_17_NO_3_ | 283.12 | N-trans-p-coumaroytyramine | 284.1307;147.0435;119.0489 |
| 20 | 19.15 | [M+H]^+^ | 842.2507 | 842.2502 | 0.6 | C_40_H_43_NO_19_ | 841.24 | Oleracein S | 842.2553;696.2159;504.1498;342.0994;177.0539 |
| 21 | 19.21 | [M+H]^+^ | 872.2623 | 872.2608 | 1.8 | C_41_H_45_NO_20_ | 871.25 | Oleracein O isomer | 872.2638;534.1621;372.1069;339.1027;177.0541 |
| 22 | 19.44 | [M+H]^+^ | 314.1379 | 314.1387 | -2.5 | C_18_H_19_NO_4_ | 313.13 | N-cis-feruloyltyramine | 314.1390;177.0544;145.0282;121.0640 |
| 23 | 20.2 | [M+H]^+^ | 284.1282 | 284.1281 | 0.3 | C_17_H_17_NO_3_ | 283.12 | N-p-​coumaroyltyramine | 284.1308;147.0440;119.0480 |
| 24 | 20.8 | [M+H]^+^ | 314.1382 | 314.1387 | -1.5 | C_18_H_19_NO_4_ | 313.13 | N-trans-feruloyltyramine | 314.1382;177.0543;145.0279;121.0648 |
| 25 | 21.24 | [M+H]^+^ | 344.1485 | 344.1492 | -2.2 | C_19_H_21_NO_5_ | 343.14 | N-trans-Feruloylmethoxytyramine | 344.1445;177.0547;145.0284;117.0329 |
| 26 | 23.39 | [M+H]^+^ | 296.0917 | 296.0917 | -0.1 | C_17_H_13_NO_4_ | 295.08 | Oleraindole A | 147.0434;119.0493;91.0536 |
| 27 | 23.78 | [M+H]^+^ | 326.1028 | 326.1023 | 1.5 | C_18_H_15_NO_5_ | 325.10 | Oleraindole B | 177.0540;149.0606;145.0281;117.0334 |
| 28 | 26.35 | [M+H]^+^ | 403.2012 | 403.2016 | -1 | C_25_H_26_N_2_O_3_ | 402.19 | Aurantiamide | 385.1913;224.1066;152.1068;117.0700;105.0335 |
| 29 | 26.5 | [M+H]^+^ | 315.1217 | 315.1227 | -3.2 | C_18_H_18_O_5_ | 314.12 | Portulacanone A | 315.1206;297.1100;221.0794;209.0794;107.0485 |
| 30 | 26.69 | [M+H]^+^ | 345.1324 | 345.1333 | -2.5 | C_19_H_20_O_6_ | 344.13 | Portulacanone B | 345.1357;327.1206;297.0736;269.0805 |
| 31 | 27.72 | [M+H]^+^ | 331.1167 | 331.1176 | -2.8 | C_18_H_18_O_6_ | 330.11 | Portulacanone C | 331.1152;313.1057;237.0754;225.0745;107.0484 |
| 32 | 28.46 | [M+H]^+^ | 445.2135 | 445.2122 | 3 | C_27_H_28_N_2_O_4_ | 444.20 | Aurantiamide acetate | 224.1066;194.1173;117.0692;105.0327 |
| 33 | 28.75 | [M+H]^+^ | 301.1071 | 301.1071 | 0.2 | C_17_H_16_O_5_ | 300.10 | Oleracone C | 301.1044;283.0952;207.0643;195.0645;167.0325 |
| 34 | 30 | [M+H-H_2_O]^+^ | 277.2145 | 277.2162 | -6.2 | C_18_H_30_O_3_ | 294.22 | 9-oxo-10E,12Z-octadecadienoic acid | 277.2152;149.1327;135.1162;121.1013 |
| 35 | 34.72 | [M+H]^+^ | 279.2307 | 279.2319 | -4.1 | C_18_H_30_O_2_ | 278.22 | Linolenic acid | 279.2311;261.2212;243.2088;149.0231 |
